# Physician’s Suicidal Behaviours in Europe: Thoughts and Beliefs of Trainees and Early Career Psychiatrists

**DOI:** 10.1192/j.eurpsy.2025.1978

**Published:** 2025-08-26

**Authors:** U. Cikrikcili, D. G. Salas, L. Schmitz, A. Szczegielniak, A. de Arriba - Arnau, S. Rosson, A. Salaj, D. Cenci, Z. Ucar, C. Tapoi, L. Lefevre, Y. Ünal-Sayali, P. Tirlea, M. Pinto da Costa, J. L. Castroman

**Affiliations:** 1 Institute of Behavioral Neurology and Dementia Research, Magdeburg, Germany; 2 Department of Addictive Disorders, Psychiatric Services, Aargau, Brugg, Switzerland; 3 Department of Psychiatry for Affective Disorders, Sahlgrenska University Hospital, Gothenburg, Sweden; 4 Department of Psychoprophylaxis, Faculty of Medical Sciences in Zabrze, Medical University of Silesia, Katowice, Poland; 5 Department of Psychiatry, Hospital Universitari de Bellvige - Psychiatry and Mental Health, IDIBELL. CIBERSAM, Carlos III Health Institute. Department of Clinical Sciences, Faculty of Medicine and Health Sciences, Universitat de Barcelona , Barcelona, Spain; 6 East London NHS Foundation Trust, London, United Kingdom; 7 Tuzla State Hospital Psychiatry Outpatient Clinic, Istanbul, Türkiye; 8 Department of Translational Medicine, Major University Hospital of Charity, Psychiatry Institute, Eastern Piedmont University, Novara, Italy; 9 Ankara Egitim Arastirma Hastanesi Psikiyatri Klinigi, Ankara, Türkiye; 10 Prof. Dr. Alexandru Obregia Clinical Psychiatry Hospital, Bucharest, Romania; 11 CHU de Montpeller Hopital Lapeyronie, Department d’Urgences et Post-Urgences Psychiatriques, Montpeller, France; 12 Zentralinstitut für Seelische Gesundheit, Marburg, Germany; 13 Psychology & Neuroscience, Institute of Psychiatry, King’s College London, London, United Kingdom; 14 University of Santiago de Compostela , Santiago de Compostela A Coruna, Spain

## Abstract

**Introduction:**

The rising rate of suicides among physicians is a critical and concerning issue that has not garnered enough attention. Personal, social, economic factors and working conditions are complicating this problem and a holistic approach is required to work towards a solution.

**Objectives:**

The aim of this study was to investigate the views of psychiatry residents and young psychiatrists working in EPA member countries on the causes, consequences and solutions of physician suicides.

**Methods:**

Under the umbrella of EPA Suicide Prevention and Suicidology Section, a 24-question survey regarding thoughts and beliefs on physicians’s suicide was developed by a group of young researchers participating in EPA Summer School 2023. The questionnaire was disseminated through EPA Early Career Psychiatrists (ECP), European Federation of Psychiatric Trainees (EFPT) email groups, communication platforms for sub-working units, and various national psychiatric associations via their email and social media channels. The goal is to collect data over at least six months. Preliminary results will be shared with participants at the EPA 2025 Congress.

**Results:**

When the initial data is analysed, it is seen that 160 people answered the questionnaire. 69% of the participants were female, 29.4% were male, 1.2% preferred not to say. The proportion of specialists was 64.4% and 35.6% of residents. Detailed responses to the questionnaire and the suggestions of the participants will be shared in detail with the congress participants in the presentation.

**Conclusions:**

This study aims to explore the perspectives of residents and early-career specialists on an issue that is stigmatized within the medical community and often avoided in open discussions. Understanding how young physicians perceive and evaluate this issue amidst increasingly challenging living and working conditions will provide valuable insights, guiding future interventions aimed at addressing and mitigating this burden.

**Disclosure of Interest:**

None Declared

